# Gene function finding through cross-organism ensemble learning

**DOI:** 10.1186/s13040-021-00239-w

**Published:** 2021-02-12

**Authors:** Gianluca Moro, Marco Masseroli

**Affiliations:** 1 DISI - University of Bologna, Via dell’Università, Cesena (FC), Italy; 2 DEIB, Politecnico di Milano, Piazza L. Da Vinci 32, Milan, 20133 Italy

**Keywords:** Biomolecular annotation prediction, Knowledge discovery, Ensemble learning, Transfer learning, Data representation, Gene ontology

## Abstract

**Background:**

Structured biological information about genes and proteins is a valuable resource to improve discovery and understanding of complex biological processes via machine learning algorithms. Gene Ontology (GO) controlled annotations describe, in a structured form, features and functions of genes and proteins of many organisms. However, such valuable annotations are not always reliable and sometimes are incomplete, especially for rarely studied organisms. Here, we present GeFF (Gene Function Finder), a novel cross-organism ensemble learning method able to reliably predict new GO annotations of a target organism from GO annotations of another source organism evolutionarily related and better studied.

**Results:**

Using a supervised method, GeFF predicts unknown annotations from random perturbations of existing annotations. The perturbation consists in randomly deleting a fraction of known annotations in order to produce a reduced annotation set. The key idea is to train a supervised machine learning algorithm with the reduced annotation set to predict, namely to rebuild, the original annotations. The resulting prediction model, in addition to accurately rebuilding the original known annotations for an organism from their perturbed version, also effectively predicts new unknown annotations for the organism. Moreover, the prediction model is also able to discover new unknown annotations in different target organisms without retraining.We combined our novel method with different ensemble learning approaches and compared them to each other and to an equivalent single model technique. We tested the method with five different organisms using their GO annotations: *Homo sapiens*, *Mus musculus*, *Bos taurus*, *Gallus gallus* and *Dictyostelium discoideum*. The outcomes demonstrate the effectiveness of the cross-organism ensemble approach, which can be customized with a trade-off between the desired number of predicted new annotations and their precision.A Web application to browse both input annotations used and predicted ones, choosing the ensemble prediction method to use, is publicly available at http://tiny.cc/geff/.

**Conclusions:**

Our novel cross-organism ensemble learning method provides reliable predicted novel gene annotations, i.e., functions, ranked according to an associated likelihood value. They are very valuable both to speed the annotation curation, focusing it on the prioritized new annotations predicted, and to complement known annotations available.

## Background

Knowledge of gene and protein biological functions in different organisms is essential to better understand human patho-physiology and agro-food production. Multiple computational approaches have been proposed to identify gene and protein functions based on the literature or experimental data [1, 2], including methods considering various types of data, also from different organisms (e.g., [3, 4]).

*Controlled biomolecular annotations* are among the most reliable data sources conveying structural and functional characteristics of genes and proteins. Several biomolecular terminologies and ontologies are available and used to express such annotations [5, 6]; among them the Gene Ontology (GO) [7] is the most developed and used one. It describes species-independent gene and protein annotations about biological processes (BP), molecular functions (MF) and cellular components (CC), with controlled terms hierarchically related within a Directed Acyclic Graph (DAG).

Controlled biomolecular annotations are key for several computationally intensive bioinformatics analyses, including *annotation enrichment analysis* [8–10], *automatic annotation of biomedical literature* [11, 12] and *semantic similarity analysis* [13–15] of genes or proteins. They are also used for the interpretation of biomolecular test results, extraction of novel information to generate and validate biological hypotheses, and also for discovering new biomedical knowledge. All these applications rely on the high coverage and quality of existing controlled biomolecular annotations. However, particularly for new and limitedly studied organisms, the annotations are typically incomplete and may contain errors. Most annotations are computationally derived, often without an associated significance level, and only a few are reviewed by experts. Although essential for annotation quality, expert curation is very time-consuming. The availability of prioritized lists of computationally predicted annotations can considerably aid and speed the curation process. In this scenario, computational methods able to accurately predict new biomolecular annotations with an associated likelihood value are crucial.

Several techniques have been proposed for prediction of gene and protein functions, and discussed in thorough reviews [16, 17]. Given the importance of this task, two Critical Assessment of protein Function Annotation (CAFA) experiments were also held, where several different methods applied to a single dataset have been evaluated on the prediction of annotations that had been discovered later [18, 19]. Many of the proposed methods use information about the genes and proteins themselves, e.g., taking advantage of similarities between amino acid sequences or evolutionary relationships. Alternatively, the prediction of new annotations can be purely based on the analysis of known existing ones.

Common machine learning methods employed for predicting new annotations from existing ones include *decision trees* [20], *Bayesian networks* [20], *k-nearest neighbours* (k-NN) [21] and *support vector machines* (SVM) classifiers [22, 23], *hidden Markov models* (HMM) [24, 25], and biological *network analysis* [26, 27]. Additionally, latent semantic approaches have been suggested, including one based on linear algebra and *singular value decomposition* (SVD) of the gene-to-term annotation matrix [28]. This approach has been extended with subsequent improvements [29–31]. Such techniques are based on simple matrix decomposition and are thus independent of both the organism and term vocabulary considered; however, they showed low efficiency.

Further techniques based on latent semantic analysis, particularly on *latent semantic indexing* (LSI) [32], have been proposed to predict new biomolecular annotations based on available annotations; they include the *probabilistic latent semantic analysis* (pLSA) [33, 34], also enhanced with *weighting schemes* [35] (for an in-depth study on term weighting see [36]), and the *latent Dirichlet allocation* (LDA) [37, 38]. Previously, we achieved good accuracy in the prediction of gene annotations to GO terms [39] leveraging the LDA technique combined with Gibbs sampling [40]. However, the complexity of the underlying model and slowness of the training process make this technique not appropriate when the size of the considered dataset increases. Other supervised methods were proposed also for the gene annotation prediction [41, 42], although with limited predictive accuracy.

New gene annotations were also inferred by taking advantage of multiple data types or sources, also regarding different organisms [43–46]. Results were better than those obtained with similar techniques applied on a single type of data; however, this approach needs a preparatory data integration phase that adds complexity, decreases flexibility, and slows the prediction process.

Cross/inter-species gene function prediction was also proposed based on gene semantic similarity [47, 48]. This improves the interpretation of the evaluated gene set behavior across organisms and can provide higher prediction performances. Yet, these are reached by taking advantage of a-priori biological knowledge to compute the similarity among genes, instead of adopting standard neutral algebraic methods to compute the gene similarities; the latter ones make the approach independent of any specific organism and allow more general and stable results across species.

Overall, the techniques previously proposed for biomolecular annotation prediction either are general and flexible, but use a simple model that gives only limited accuracy, or improve prediction results in different ways, such as by taking advantage of a-priori biological knowledge or of a complex integrative analytic framework, or by adopting a more complex model. The latter ones are often difficult and time consuming to be suitably set up, and their prediction process is slow, particularly when a large amount of data is evaluated.

Previously, we proposed both an innovative representation of the annotation discovery problem and a random perturbation method of the available annotations [49]. As we proved, they allow taking advantage of supervised algorithms to make use of the available annotations of the genes of an organism to accurately predict novel controlled annotations for the same genes, providing a likelihood value associated with each predicted annotation. Then, we considered innovative approaches proposed in machine learning about domain adaptation [50, 51] and cross-domain transfer learning [52–56]. Taking inspiration from comparative genomics, we evaluated the feasibility and performance of applying these approaches to predict new functionalities for the genes of an organism based on the known annotations for the genes of a different organism [57]. We demonstrated that, using our proposed classification-based method, the available knowledge about a more studied organism can be used to enhance the prediction models related to a less studied organism (conversely, using for the model training a less studied organism typically provides worst annotation predictions).

Here, we propose the enhancement of that transfer learning approach with *ensemble learning*, in order to reliably predict with high precision across organisms novel gene annotations, with an associated likelihood value for their prioritization. In ensemble learning, multiple different models are trained on the same data and their predictions are combined (e.g., by voting or averaging), so that they are treated as a single model. Ensemble learning has been previously proposed in the context of gene and protein function prediction, but alone and on limited sets of data of a single organism [58–63]; we innovatively apply this approach together with transfer learning involving data from multiple organisms.

We compare different ensemble classification model methods with each other and with the equivalent single model technique on different gene annotation datasets of five eukaryotic organisms (*Homo sapiens*, *Mus musculus*, *Bos taurus*, *Gallus gallus* and *Dictyostelium discoideum*), showing that ensemble methods provide better results and also offer the ability to customize the trade-off between the number of predicted novel annotations and their precision. Furthermore, we apply our last enhanced technique to predict and prioritize the most probable missing GO annotations of the genes of the organisms in the Entrez Gene database [64]. Finally, we also develop the Gene Function Finder (GeFF) Web application, to enable any user to efficiently generate such predicted annotations according to the user-selected ensemble method and defined parameters, and to easily browse and download both the predicted new gene GO annotations and the available known ones used for the prediction.

## Methods

### Datasets

For comparison with previous works, we used the same datasets that they employed. To do so, we took advantage of the Genomic and Proteomic Data Warehouse (GPDW) [65–67], to retrieve multiple gene annotation sets of different organisms. GPDW integrates several sources of genomic and proteomic controlled annotations for many species, providing application programming interfaces (APIs) to automatically retrieve them. GPDW stores different outdated versions of the contained annotations, which we used to quantitatively evaluate our novel prediction method and compare it with previous proposed methods. In particular, we used two temporally different versions of the GO annotations available in the GPDW for the genes of the selected organisms: an older version, as of July 2009, and a more recent one, as of March 2013, which were used in the evaluation of previous works.

In our datasets, in addition to the annotations explicitly stored in GPDW regarding the specific GO terms, we also considered annotations to the ancestors of the same terms, according to the GO hierarchical structure. GO uses a set of *evidence codes* to label each annotation based on how it was produced, including “Inferred from Electronic Annotation” (IEA) and “No biological Data available” (ND) codes, used for annotations without human expert review. Taking advantage of this, we distinguish between less reliable annotations, which are labelled with IEA and/or ND evidence codes only, and reliable annotations, which are labelled with at least one different code. The dataset used to train the models only includes July 2009 reliable curated annotations (*A*_09_ in Table 1), while the dataset used for evaluation includes all the March 2013 available annotations (*At*_13_ in Table 1). Table 1 reports the counts of genes, terms and annotations for each organism involved in the evaluation.

**Table 1:** Counts of GO annotations (*A*) and involved genes (*G*) and GO terms (*T*) used for the evaluation of our cross-organism approach. Only genes and GO terms shared between the July 2009 and March 2013 versions are counted. Both most specific and implicit annotations are counted. As annotations of the July 2009 version, only the more reliable curated annotations used for model training (*A*_09_) are reported. As annotations of the March 2013 version, both curated (*A*_13_) and total (*At*_13_) annotations are reported; the latter ones are those used for model evaluation

	*Homo*	*Mus*	*Bos*	*Gallus*	*Dictyostelium*
	*sapiens*	*musculus*	*taurus*	*gallus*	*discoideum*
*G*	9,937	9,265	646	321	1,762
*T*	3,322	3,366	749	403	1,016
*A*_09_	345,259	319,402	21,305	8,846	65,421
*A*_13_	353,679	606,239	26,194	11,339	63,621
*At*_13_	955,341	826,033	47,237	17,744	118,695

### Cross-organism supervised prediction algorithm

In this section we illustrate how we address the prediction of novel gene annotations of an organism based on its available annotations as a supervised problem, in which it is possible to train a supervised prediction model to do so. Furthermore, we show how to transfer knowledge available for a different more studied organism in order to improve the prediction precision on a less studied one. Then, in the next section we describe our ensemble approach, innovatively combined with the here illustrated ones.

#### Data representation for supervised prediction

We first define a set $\mathcal {T}=\left \{t_{1},\ldots,t_{n}\right \}$ of controlled GO terms to be considered. Then, for each organism with a set of genes {*g*_1_,…,*g*_*m*_}, we define its *annotation matrixA* as a *m*×*n* binary matrix, where *A*[*i*,*j*]=1 if it exists an annotation that associates gene *g*_*i*_ to GO term *t*_*j*_,*A*[*i*,*j*]=0 otherwise. The discovery of unknown annotations entails predicting, for each gene-term pair (*g*_*i*_,*t*_*j*_) for which an annotation does not currently exist (*A*[*i*,*j*]=0), whether it is likely or not that *g*_*i*_ and *t*_*j*_ are actually associated, i.e., whether or not *A*[*i*,*j*] should be 1.

The goal of the annotation prediction can be viewed as the discovery of the annotations that are missing in an outdated version of the annotation matrix *A* and will be present in a more updated version of it. This problem can be modelled as a *supervised multi-label classification* problem [68], where terms alternatively act as features or labels. Specifically, for each term $t_{c}\in \mathcal {T}$, we train a specific binary classifier to predict whether a gene is associated with *t*_*c*_ (the class-term) from the associations with all other terms in $\mathcal {T}$, used as predictive features. By using two versions of the annotation matrix, an outdated one *A*_0_ with less annotations and an updated one *A*_1_, it is possible to create a dataset to use for the supervised model training. Each classifier is trained using values for the class-term in the updated matrix as the prediction goals, and values for all other terms in the outdated matrix as features.

Considering that outdated versions of an annotation matrix could be unavailable, we have shown in [49] how to generate an artificial older version of the matrix by randomly removing some annotations from its current version. Given a current matrix *A*_1_,*A*_0_ is a copy of it where each element equal to 1 in *A*_1_ is set to 0 with a preset probability *p*. After generating *A*_0_, a *perturbation unfolding* process further refines it to remove annotations that, after the perturbation, are no longer consistent with the GO hierarchical structure.

#### Prediction likelihood

Each binary classification model, trained as above considering a class-term *t*, can be used to estimate, given the annotation profile of a gene *g* to other terms, whether or not *g* is potentially annotated to *t*. Rather than giving a binary response 1 (yes) or 0 (no), commonly employed models return a value laying between 1 and 0, which expresses the degree of confidence in the prediction: the more the value is close to 1, the more the classifier is confident about the prediction. In this context, the value *p*(*g*,*t*) reported by a model indicates the probability, or *likelihood*, of gene *g* to be annotated to term *t*.

Answers from models independently trained on different class-terms may violate the *True Path Rule*, which states that if a gene *g* is annotated to a term *t*, it must be also annotated to any ancestor of *t* in the GO term hierarchy [69]. To get likelihood values consistent with this rule, we apply two post-processing steps to each “raw” likelihood value *p*(*g*,*t*) given by the models. First, for each gene *g* and each term *t*, we average the gene-term annotation likelihood with the mean likelihood of the annotations of *g* to the ancestors of *t*:

1 $$ p^{H}(g,t)=\frac{\frac{\sum_{t_{a} \in {ancestors}(t)}p(g,t_{a})}{|{ancestors}(t)|} + p(g,t)}{2}  $$

Then, starting from leaf GO terms, we fix violations of the rule in case present; we do so by computing the final likelihood *l*(*g*,*t*) for each potential gene-term annotation as the maximum average likelihood of *g* being associated with either *t* or one of its descendants:

2 $$ l(g,t)=\max \left\{p^{H}(g,t),\max_{t_{d} \in descendant(t)} \left\{ p^{H}(g,t_{d})\right\} \right\}  $$

Thus, for any gene *g* and any pair of GO terms *t*_*a*_ and *t*_*d*_ where the former is an ancestor of the latter, *l*(*g*,*t*_*a*_)≥*l*(*g*,*t*_*d*_) holds.

In order to get a final list of predicted gene-term annotations, we consider only (*g*,*t*) pairs for which there are no already known annotation (i.e., *A*_1_(*g*,*t*)=0) and prioritize them by their likelihood *l*(*g*,*t*) given by the prediction model. By setting a likelihood threshold *ρ*, we can define a subset of “reliable” predictions whose likelihood is at least *ρ*.

#### Cross-organism approach

The proposed method relies on a training phase and thus its precision highly depends on the available training annotation matrix. When only a small set of known annotations (i.e., a very sparse annotation matrix) is available for the organism whose annotations we want to predict, the trained model may be not very effective. To overcome this issue, in [57] we proposed a cross-organism method in which the prediction model is trained on a well-studied and better-known organism (called *source*), and then it is used to predict novel unknown annotations of a less studied *target* organism. Such approach is based on annotation terms co-occurring in both source and target organisms, independent of the specific genes they annotate. This has proven to be effective in predicting annotations for less known organisms, for which scarce amounts of data would be otherwise available to train accurate models.

The cross-organism learning method requires the selection of genes and terms useful to predict novel gene annotations for the target organism. The set of terms $\mathcal {T}$ considered in the prediction model is the intersection $\mathcal {T}_{S}\cap \mathcal {T}_{T}$ of terms present in the source ($\mathcal {T}_{S}$) and target ($\mathcal {T}_{T}$) organisms, while the genes of the source organism used to train the model are those having at least 5 annotations to the terms of $\mathcal {T}$.

### Ensemble learning method

In this section we describe our ensemble approach, originally combined with the cross-organism supervised prediction method.

#### Supervised learning algorithm

Ensemble learning methods are based on sets of machine learning algorithms whose decisions are combined in some way to improve the performance of the overall system [70]. The key concept of ensemble learning is that no single algorithm can claim to be uniformly superior to any other; hence, an ensemble classifier can have overall better performance than the individual base classifiers it combines.

Most popular supervised learning techniques include Support Vector Machines, decision trees, and Nearest Neighbors. To create our prediction model we use the Random Forest (decision trees) algorithm, since several works [70, 71] have shown that decision trees tend to generate different classifiers even with small changes in the training data and are therefore suitable candidates for the base learners of an ensemble system. Furthermore, results in [72] show that for the considered task the Random Forest classifier achieves better performance with respect to Support Vector Machine with radial basis kernel and k-Nearest Neighbors. Anyway, our proposed ensemble approach can be equally used with any supervised learning algorithm.

#### Ensemble approach

One of the most common way to build ensembling approaches is based on the injection of randomness in training data [73]. We make use of it to build our ensemble learning method. Starting from the known annotation matrix of the source organism *As*_1_, we create *n* different randomly perturbed versions of it, by changing the perturbation random seed but keeping the same perturbation probability; hence, we artificially create *n* distinct training matrices ${As}_{0}^{i}$, with 1≤*i*≤*n*, which we use to train *n* different prediction models. Then, each prediction model *m*_*i*_ is applied to the known annotation matrix of the target organism *At*_1_ to built a prediction matrix ${At}_{2}^{i}$. In this way, the method gives *n* different likelihoods for each possible gene-term association in the target annotation matrix, allowing for multiple voting approaches. To produce the final predicted novel annotations we propose two approaches:

- *Average score* (AVG): the final probability *l*(*g*,*t*) of the gene *g* to be annotated to the term *t* is the average of all predicted likelihoods given by the single models: $l(g,t)=\sum _{i=1}^{n}{\frac {l_{i}(g,t)}{n}}$. The final annotations predicted are those with probability greater than a defined threshold *ρ*.
- *Voting x out of n* (∩_*x*/*n*_): A missing gene-term annotation is considered as a new predicted annotation if at least *x* out of *n* single models have predicted the annotation with probability greater than a defined threshold value *ρ*. Notably, *x*=1 indicates the union of all the predictions done by any of the single models, and symmetrically *x*=*n* means their intersection. The choice of the most suitable value of *x* can be optimized for the specific task.

**Fig. 1:**
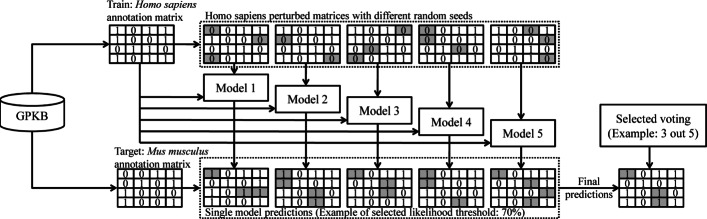
Process of the proposed ensemble method. The annotation matrices of the source (*Homosapiens*) and target (*Musmusculus*) organisms are extracted from the GPKB; with *n* (*n*=5 in the example) different perturbations on the former one, *n* matrices are created and used to train *n* different prediction models, each of them is then applied on the target annotation matrix; out of the single model predictions, a voting approach (3 out of 5 in the example) selects the final predicted annotations

In Fig. 1 a simple example of the proposed ensembling process is shown, which uses a *3 out of 5* voting approach: first, the source and target known annotation matrices are extracted from the GPKB; then, the source matrix is perturbed *n*=5 times using different random seeds. This leads to artificially create 5 different annotation matrices that, together with the known one, are used to build 5 different prediction models. Each model predicts what 0 in the target annotation matrix are likely to be 1 - in the illustrated example all the predictions with probability (i.e., likelihood) greater than 70%. Finally, as an example, only the associations predicted by at least 3 models are provided as final predicted annotations.

## Experimental evaluation results

In order to assess the quantity and especially the exactness of the novel annotations predicted by our method, we run tests on different target organisms. We summarize here the effects obtained by varying the parameters of the method and show the achieved results.

As *Homo sapiens* is the organism for which most annotations are available, we took advantage of them to create the annotation matrix used to train classification models. Where not stated otherwise, training annotations did not include those with the IEA evidence code only. Out of the evolutionary divergent eukaryotic species that were previously taken into account in the Reactome pathway knowledge base project for a similar orthology inference strategy [74], we used other four organisms as prediction targets, namely *Mus musculus*, *Bos taurus*, *Gallus gallus* and *Dictyostelium discoideum*, which differ for their number of genes, functions and known annotations, as shown in Table 1, and for their evolutionary distance from *Homo sapiens*.

For the training matrix, in both cases with and without IEA-only annotations, 5 different randomly perturbed versions were extracted for each considered perturbation probability *p* ranging between 1% and 20%. Thus, for each of them we obtained 5 different models, which were combined into an ensemble using either the average (AVG) of the likelihood scores given by the component models or the voting *x* out of 5 (∩_*x*/5_, with *x*∈{1,2,3,4,5}) approach, which considers annotations predicted by at least *x* out of the 5 models. Results obtained with ensembles were compared against a single model (SM), whose performance was estimated as the average performance of the same 5 models considered separately. All these cases were tested for different values of the prediction likelihood threshold *ρ* ranging between 0.05 and 0.95.

Our method has the goal of ensuring that most of our predicted annotations are correct, while predicting as many novel annotations as possible. This is driven by the high biological interest of having prioritized annotations as reliable as possible, in spite of missing some possible annotations, rather than of identifying a greater number of potential annotations, but including many probable incorrect ones, which are then costly and time consuming to be experimentally validated. Accordingly, to evaluate our method we consider two key performance measures: the *number of predictions* indicates the total count of novel likely gene-term associations predicted by a model for a target organism, while the *precision* indicates the percentage of how many of such predictions are present as actual annotations in the up-to-date version of the target known annotation matrix used for validation. It is worth noting that these two performance measures fully and exactly evaluate the goal of our prediction method, without the need of other additional measures typically used together with them, which conversely evaluate other different aspects of a prediction that are not relevant for our goal. Furthermore, in evaluating the obtained precision values it should be kept in mind that, despite being true, predicted annotations may not be in the newer annotation matrix just because they have not yet been discovered, given the potentially high number of still unknown annotations for a target organism; thus, the precision values that can be calculated could underestimate the real precision of the prediction method.

**Fig. 2:**
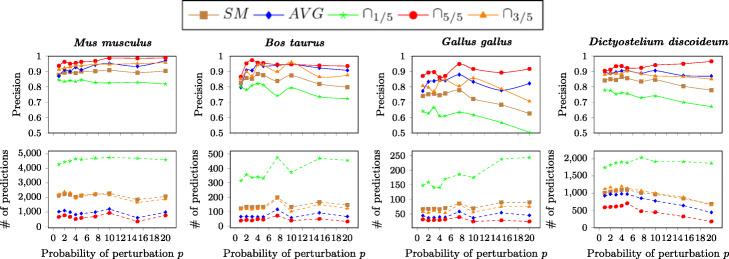
Evaluation results by varying the perturbation percentage. Results obtained by varying the perturbation percentage of the *Homosapiens* training annotation matrix and using *Random Forest* as supervised algorithm and the likelihood threshold *ρ*=0.8. *SM* is the single model method, proposed in [57]; *AVG* considers the average of the likelihood scores given by the models inferred from five different perturbation random seeds; ∩_1/5_ considers the union of the predictions from the five models; ∩_5/5_ considers the intersection of the predictions from the five models; ∩_3/5_ considers those predictions from three out of the five models

We first analyze the effect of varying the perturbation probability *p* and the ensemble classification method. Figure 2 reports, for the four considered target organisms, the variation of both the count of novel predicted annotations and their precision for the prediction likelihood threshold *ρ* = 0.8.

The ∩_5/5_ ensemble method, i.e., the intersection of single models, being the most selective one, is at all times the most precise one, at the cost of a lower number of obtained predictions. In comparison, the ∩_1/5_ method, i.e., the union of single models, provides 2.5 to 15 times more predictions, but its precision drops from above 90% to values ranging from 50% to 85%. Both the precision and the number of predictions of other methods lie between ∩_5/5_ and ∩_1/5_. We can see the advantage of using ensembles over single models by comparing results of SM with ∩_3/5_. While both provide a very similar number of predicted annotations, the precision of the latter one is always superior, with an absolute difference between 1.5% and 13%. Furthermore, the SM precision only overcomes that of ∩_1/5_, which in turn predicts a considerably higher number of new candidate annotations, generated by at least one of the single models in the ensemble. This confirms the goodness of the ensemble approach, and that perturbing with different random seeds the training matrix leads to the creation of different models that generate different predictions and likelihoods.

When changing the perturbation probability *p*, the variation in results is mostly irregular. A trend noticeable in some cases is that the difference in precision and number of predictions between the models tends to increase for higher values of *p*. This can be explained by the fact that single models in the ensembles tend to be more different and then more likely to give different answers. In the following, we assume *p*=10*%* by default, which gives reasonable model diversity, precision and number of predictions.

**Fig. 3:**
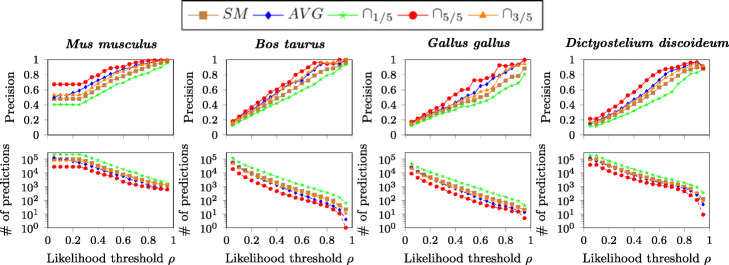
Evaluation results by varying the likelihood threshold. Results obtained by varying the value of the likelihood threshold and using *Random Forest* as supervised algorithm, trained on perturbed versions of the *Homosapiens* annotation matrix with perturbation percentage *p*=10*%*. *SM* is the single model method, proposed in [57]; *AVG* considers the average of the likelihood scores given by the models inferred from five different perturbation random seeds; ∩_1/5_ considers the union of the predictions from the five models; ∩_5/5_ considers the intersection of the predictions from the five models; ∩_3/5_ considers those predictions from three out of the five models

In Fig. 3 we see instead the effect of varying the likelihood threshold *ρ*. Intuitively, by increasing the value of *ρ* we get fewer but more precise predictions. Generally, using the most precise ∩_5/5_ ensemble method, values of *ρ* above 0.75 guarantee a precision close to 90%. This parameter can be effectively used to tune the desired trade-off between getting more predictions and obtaining more precise answers.

### Comparison of learning algorithms

We compared the observed performances of the Random Forest algorithm, used to obtain all the presented results, with those of the k-Nearest Neighbors algorithm. Other than the number of predicted annotations and their precision, in this comparison we also considered the average depth of the terms of the predicted annotations within the Gene Ontology taxonomy. Table 2 reports these performance measures for the two algorithms for every considered target organism and ensemble method type. Results indicate that generally the Random Forest classifier provides more precise models; whereas, the k-Nearest Neighbors classifier generally provides predicted annotations with an higher level of the involved annotation terms, meaning that predictions concern more specific terms in the Gene Ontology hierarchy, but the precision of such predictions is lower in most cases (up to 50% less than Random Forest). Similarly, models with lower number of predicted annotations (mostly from ∩_5/5_ and AVG ensemble methods), besides being often more precise, also generally provide annotations to terms with an higher level in the GO taxonomy, thus related to more specific functions and hence more valuable.

**Table 2:** Comparison of performances of Random Forest (RF) and k-Nearest Neighbors (k-NN) classifiers in terms of number of predicted annotations (*N*), their precision (*Pr*) and the average level ($\overline {L}$) of predicted annotation terms in the Gene Ontology DAG (when the term of a predicted annotation belongs to multiple Gene Ontology levels, only its lowest level was considered). SM is the single model method; AVG considers the average of the likelihood scores given by the models inferred from five different perturbation random seeds; ∩_*x*/5_ considers those predictions from x out of the five models. Probability of perturbation *p* and likelihood threshold *ρ* were set to their respective default values *p*=10*%* and *ρ*=0.8

		RF			k-NN		
**Target**	Ensemble method	*N*	*Pr*	$\overline {L}$	*N*	*Pr*	$\overline {L}$
*Mus*	SM	2,285	0.908	1.604	2,841	0.803	1.864
*musculus*	AVG	1,204	0.952	1.799	1,227	0.817	2.487
	∩_1/5_	4,753	0.826	1.736	6,378	0.704	1.888
	∩_2/5_	2,896	0.916	1.653	3,380	0.836	1.826
	∩_3/5_	2,157	0.947	1.569	2,396	0.911	1.734
	∩_4/5_	1,764	0.973	1.491	1,499	0.937	1.774
	∩_5/5_	932	0.987	1.626	552	0.955	2.317
*Bos*	SM	132	0.874	2.721	123	0.657	2.835
*taurus*	AVG	57	0.947	3.037	44	0.568	3.000
	∩_1/5_	373	0.794	2.544	355	0.625	2.725
	∩_2/5_	173	0.925	2.831	155	0.710	2.864
	∩_3/5_	100	0.960	2.854	62	0.726	3.022
	∩_4/5_	60	0.967	2.931	32	0.656	3.238
	∩_5/5_	37	0.946	3.143	13	0.462	3.500
*Gallus*	SM	69	0.721	2.701	50	0.534	3.255
*gallus*	AVG	36	0.833	3.367	29	0.690	3.800
	∩_1/5_	175	0.617	2.157	137	0.416	2.509
	∩_2/5_	88	0.682	2.700	55	0.564	3.290
	∩_3/5_	56	0.857	2.958	31	0.742	3.652
	∩_4/5_	38	0.895	3.324	17	0.765	4.308
	∩_5/5_	24	0.917	3.545	11	0.909	5.000
*Dictyostelium*	SM	966	0.846	2.522	1,029	0.718	2.651
*discoideum*	AVG	773	0.906	2.500	869	0.794	2.733
	∩_1/5_	1,917	0.741	2.454	2,108	0.574	2.518
	∩_2/5_	1,334	0.833	2.531	1,233	0.737	2.664
	∩_3/5_	997	0.872	2.517	858	0.830	2.718
	∩_4/5_	760	0.905	2.529	622	0.883	2.703
	∩_5/5_	444	0.941	2.555	326	0.951	2.900

### Impact of IEA-only annotations

In our experiments, by default, annotations used for model training did not include those with only the IEA (*Inferred from Electronic Annotation*) evidence code, denoting annotations obtained by automated methods. To evaluate this choice, we compared the performances of prediction models obtained either including or not annotations with only the IEA evidence code in the training set.

**Fig. 4:**
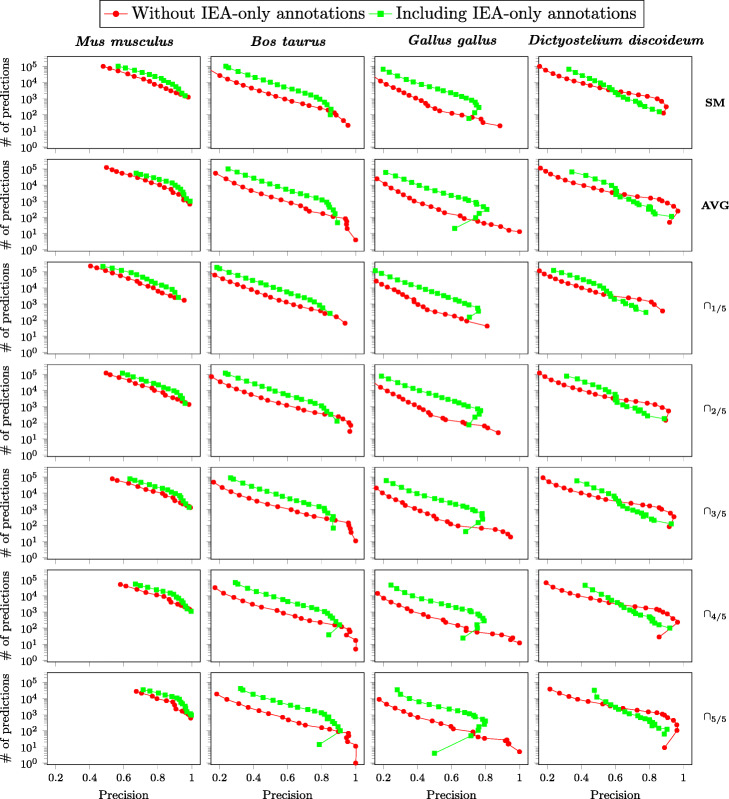
Prediction model precision vs. number of predictions. Precision (X axis) and number of predictions (Y axis) of prediction models trained on *Homosapiens* known annotations, either excluding or including IEA-only annotations, and tested on four target organisms (one per column of plots) on each of the considered ensemble methods (one per row of plots). Each point in the plots represents a test result obtained with a different value of the predicted annotation likelihood threshold *ρ*, ranging from 0.05 to 0.95; leftmost points in each plot correspond to values lower than *ρ*

Figure 4 shows how precision and number of predictions vary in the target organisms according to the ensemble method and the annotation likelihood threshold *ρ*. Results suggest that using also IEA-only annotations in model training in most cases provides an improvement in both the number of predictions and their precision.

However in some cases, notably in *Gallus gallus* and *Dictyostelium discoideum* with more selective ensemble methods such as ∩_5/5_, raising the likelihood threshold *ρ* on models trained with also IEA-only annotations causes a notable loss of precision. Conversely, performances of models trained without IEA-only annotations exhibit a more predictable trend in the precision as the likelihood threshold varies.

### Comparison with previous works

As we used datasets that were also employed in previous works [75, 76], where multiple methods to predict novel gene annotations from known ones were compared, we could assess the relevance of our approach by comparing its precision performances on these datasets with the precisions previously published of such several other methods applied on exactly the same datasets.

Both [75] and [76] used the *Bos taurus* July 2009 and March 2013 gene GO annotation datasets (and [76] used also the correspondent two *Gallus gallus* datasets) from the Genomic and Proteomic Data Warehouse as we did: the 2009 dataset as prediction input, the 2013 one to evaluate the obtained results.

In [75], the authors compared three latent semantic analysis computational algorithms, with or without different weighting schemes: the Latent Semantic Indexing (LSI) [77], probabilistic Latent Semantic Analysis (pLSA) [78, 79], and Semantic IMproved Latent Semantic Analysis (SIM) [80], an extension of LSI with a clustering step on the evaluated genes. Without any weighting scheme, these three algorithms showed similar performances. Using weighting schemes (NTN: No transformation - Term weight - No normalization, NTM: No transformation - Term weight - Maximum, or ATN: Augmented - Term weight - No normalization) generally improved their performances, in particular for LSI and SIM in combination with the ATN weighting scheme; the SIM method coupled with the ATN weighting scheme resulted the one with better performance. However, even the latter one’s performance resulted greatly lower than the one of our proposed approach: 32.2% of predicted annotations confirmed (precision 0.322) vs. 46.2% (precision 0.462) for our worst k-Nearest Neighbors ensemble method and 96.7% (precision 0.967) for our best Random Forest ensemble method (Table 3).

**Table 3:** Comparison of performances (precisions) of Random Forest (RF) and k-Nearest Neighbors (k-NN) classifiers with performances of other methods in published works ([75, 76]) over the same datasets. SM is the single model method; AVG considers the average of the likelihood scores given by the models inferred from five different perturbation random seeds; ∩_*x*/5_ considers those predictions from *x* out of the five models. For RF and k-NN evaluations, probability of perturbation *p* and likelihood threshold *ρ* were set to their respective default values *p*=10*%* and *ρ*=0.8

Classifier/Work	Method	*Bostaurus*	*Gallusgallus*
RF	SM	0.874	0.721
	AVG	0.947	0.833
	∩_1/5_	0.794	0.617
	∩_2/5_	0.925	0.682
	∩_3/5_	0.960	0.857
	∩_4/5_	0.967	0.895
	∩_5/5_	0.946	0.917
k-NN	SM	0.657	0.534
	AVG	0.568	0.690
	∩_1/5_	0.625	0.416
	∩_2/5_	0.710	0.564
	∩_3/5_	0.726	0.742
	∩_4/5_	0.656	0.765
	∩_5/5_	0.462	0.909
[75]	LSI	0.260	-
	LSI-NTN	0.248	-
	LSI-NTM	0.192	-
	LSI-ATN	0.282	-
	SIM	0.190	-
	SIM-NTN	0.206	-
	SIM-NTM	0.240	-
	SIM-ATN	0.322	-
	pLSA	0.206	-
	pLSA-NTN	0.212	-
	pLSA-NTM	0.202	-
	pLSA-ATN	0.162	-
[76]	tSVD (LSI)	0.210	0.097
	SIM1 (SIM)	0.157	0.103
	SIM2	0.197	0.097
	pLSA	0.277	0.233
	LDA	0.217	0.127
	AE	0.397	0.397

In [76], the authors compared the same three algorithms as in [75] (i.e., LSI, also known as truncated Singular Value Decomposition (tSVD), pLSA, and SIM, which they call SIM1) and other three state-of-the-art algorithms: Autoencoder Neural Networks (AE) [81], Latent Dirichlet Allocation (LDA) [82], and another extension of LSI, with term-term similarity weights besides gene clustering (named SIM2) [80]. On both the *Bos taurus* and *Gallus gallus* datasets, overall the tSVD-based methods (tSVD, SIM1, SIM2) achieved similar performances, and LDA resulted comparable to them. AE was consistently the best, with its performance improved on average by +43.3% to +70.0% with respect to the performance of pLSA, which performed slightly better than the other considered methods. Yet, on average only 39.7% of the AE predicted annotations resulted confirmed (precision 0.397), much less than with our approach (Table 3).

All comparisons confirmed the relevance of our proposed cross-species ensemble approach, which greatly outperformed all other considered methods providing predicted gene GO annotations with much higher precision, even when it predicted a relevant number of annotations. This was achieved thanks to our novel proposal of coupling an ensemble approach with a supervised method and a richer annotation matrix to train the models.

## Implementation and Web application

We implemented the described method in Java programming language, using the WEKA machine learning software [83, 84] to generate Random Forest and k-Nearest Neighbors prediction models.

Furthermore, we developed a Python-based Web application, named GeFF (Gene Function Finder), to provide an intuitive interface both to easily predict novel annotations for selected organisms and to browse either the novel predicted annotations generated or the known annotations used to predict new ones. GeFF and its documentation are publicly available at http://tiny.cc/geff/.

The GeFF Web interface allows the user to get all known or new predicted gene GO annotations for a desired organism among the several available ones, optionally limiting the retrieved annotations to those of one or more selected genes. Moreover, the user can specify the type of ensemble approach and the likelihood threshold to use to define the novel annotations predicted. As output, the GeFF Web system gives a list of known or predicted gene GO annotations, which can also be exported to a comma-separated values (CSV) file for further easy processing.

### GeFF Web application engineering

To predict the annotations for a given target organism, our approach requires computing five different prediction models, each consisting of hundreds or usually thousands of individual decision trees models, one for each annotation term included as a feature in the specific prediction model. Clearly, all such computations cannot be performed in real time. Thus, for all the available organisms, we pre-computed all the predicted gene GO annotation likelihood values for each of the single models considered, and stored them in a database along with the known annotations; this then allows showing quickly in the GeFF Web application the predicted annotations according to the user-specified ensemble model and parameter values. In order to provide the annotations predicted by the ensemble approach, at user request time only the combination of the prediction models is efficiently calculated according to the parameter values given by the user. Specifically, for every target organism, the GeFF database stores the likelihood of each potential gene-term association estimated by each of the five models trained on differently perturbed versions of the known annotation matrix. Every time the user requests predictions for an organism, the GeFF application efficiently combines the five models according to the user-selected type of ensemble to consider (AVG or ∩_*x*/5_) and filters the results according to the user-indicated likelihood threshold *ρ*, providing the ensemble predicted annotations within the GeFF Web interface.

**Fig. 5:**
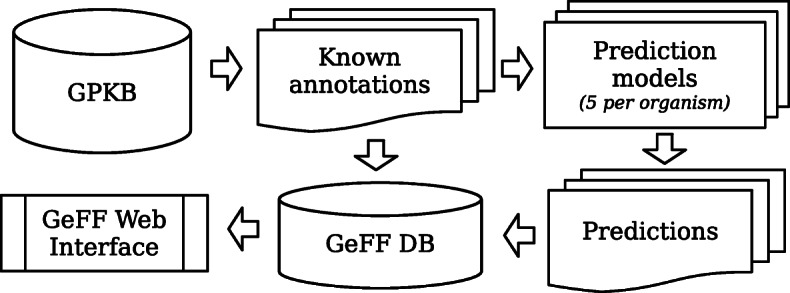
Pre-calculation process of predicted annotations managed in the GeFF Web application

**Fig. 6:**
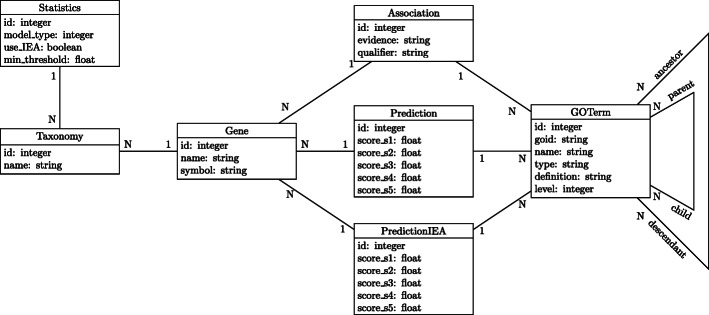
Logical schema of the relational database created for the GeFF Web application. It stores all the known and predicted annotations used by the created Web application

To support the GeFF Web application, we created a computational framework that off-line automatically downloads the known annotations from GPKB, generates the predictive models for each organism of such annotations and stores all known and predicted annotations into a specifically created database, which is then used by the GeFF Web application. The flow of this process is illustrated in Fig. 5, while Fig. 6 shows the logical schema of the created relational database populated by this process and used by the GeFF Web application.

## Discussion and conclusions

We developed a novel cross-organism ensemble learning approach and originally applied it to automatically infer new unknown gene GO annotations of target organisms. Taking advantage of the knowledge learned from a source organism better studied, the method discovers unknown gene GO annotations of another target organism evolutionarily related and less studied, namely with a smaller number of known annotations. To our knowledge, this is a first effective ensemble learning method to improve the knowledge of less studied organisms by exploiting available annotations of a better studied one.

Our approach takes advantage of both an innovative representation of the annotation discovery problem, which allows us to address it as a supervised problem despite the unsupervised nature of the task, and a random perturbation method of the source organism available annotations, as a base for building multiple models for ensemble learning. The combination of transfer and ensemble learning and the use of different random perturbations of the gene known annotations as the base for building multiple models for ensemble learning are the main conceptual advances of our work over previous works. Notably, our approach provides ranked lists of predicted gene annotations that describe novel gene functions and have an associated likelihood value. Thus, they are very valuable both to complement available annotations, for better coverage of the many gene functionalities in biomedical knowledge analyses, and to quicken the annotation curation process, by focusing it on the prioritized novel annotations predicted.

We assessed quantity and exactness of the novel annotations predicted with our ensemble learning approach using different ensemble learning methods on different gene annotation datasets of five evolutionarily related organisms. We compared them with each other and with those from the equivalent single learning model. Results showed the annotation prediction improvement of the cross-organism ensemble learning approach with respect to the single model, regardless of the evolutionary distance between the considered source and target organisms, and the reliability of the novel gene annotations that it can discover.

Comparison with results from previously proposed methods for novel gene annotation prediction based on known ones, which do not take advantage of cross-species or ensemble learning, showed the great improvement in precision of the new annotations that our method predicts on the same datasets. Furthermore, thanks to the transfer learning approach, our method is able to provide potential annotations for organisms that are less studied and generally not considered in experimental evaluations of other methods. Thus, despite the high amount of work previously done on gene function prediction, our innovative approach proves significant in reliably providing novel annotations particularly for those organisms with still few annotations available.

The GeFF Web application that we developed allows the easy use of our approach to predict new gene GO annotations for several organisms according to the user-selected ensemble method to use and a few user-definable parameter values; they enable the user to tune the desired trade-off between number of predictions obtained and their precision. Furthermore, the GeFF Web application eases browsing and retrieval of both the predicted annotations and the available known ones used for the prediction.

Despite our focus on Gene Ontology annotations of genes, the proposed approach can be equally applied to protein annotations. Additionally, it is not bound to Gene Ontology annotations, but it can be applied to any type of controlled annotations, from an ontology or even a flat terminology.

While we made use of well-established machine learning algorithms, additional efforts to further improve the quality of the provided predictions may be made by testing different methods. Approaches based on deep neural networks, garnering widespread attention in the machine learning community in the latest decade, might be good candidates for the genomic prediction task. Such approaches may have strong capabilities in discovering and modeling latent associations between terms, thus boosting the precision in the prediction of unknown annotations.

## Data Availability

A Web application to browse both input annotations used and predicted ones, choosing the ensemble prediction method to use, is publicly available at http://tiny.cc/geff/.

## Abbreviations

API: Application programming interface
AVG: Average score
BP: Biological processes
CC: Cellular components
CSV: Comma-separated values
DAG: Directed acyclic graph
GeCo: Genomic computing
GeFF: Gene function finder
GO: Gene ontology
GPDW: Genomic and proteomic data warehouse
GPKB: Genomic and proteomic knowledge base
HMM: Hidden Markov models
IEA: Inferred from electronic annotation
k-NN: K-nearest neighbour
LDA: Latent Dirichlet allocation
LSI: Latent semantic indexing
MF: Molecular functions
ND: No biological data available
pLSA: Probabilistic latent semantic analysis
SM: Single model
SVD: Singular value decomposition
SVM: Support vector machines
∩_*x*/5_: voting *x* out of 5 (with *x*∈{1,2,3,4,5}) ensemble approach.
